# The F‐words for childhood development in community‐based physical activity programmes for children with physical disabilities: A scoping review

**DOI:** 10.1111/dmcn.70306

**Published:** 2026-05-11

**Authors:** Belinda Munroe, Sarah Ashcroft, Suzanne Lock, Kylie Turner, Belinda Arnold, Sarah Reedman

**Affiliations:** ^1^ Frame Running Wollongong Wollongong NSW Australia; ^2^ School of Nursing, Faculty of Science Medicine and Health, University of Wollongong Wollongong NSW Australia; ^3^ Queensland Cerebral Palsy and Rehabilitation Centre, Faculty of Health, Medicine and Behavioural Sciences, The University of Queensland Brisbane QLD Australia; ^4^ Suzy Lock Kids Physio Wollongong NSW Australia; ^5^ Cerebral Palsy Alliance, The University of Sydney Sydney NSW Australia

## Abstract

**Aim:**

To identify intervention ingredients used to address the F‐words for childhood development in community‐based physical activity programmes for children with physical disabilities, the programme outcomes, and the strength of available evidence.

**Method:**

A scoping review was conducted guided by the JBI Manual for Evidence Synthesis. Electronic database and hand searches of international literature identified studies that evaluated community‐based physical activity programmes for children with a physical disability with impaired gross motor skills (e.g. sitting, walking) ranging from mild limitations in speed, balance, and coordination to severe impairment requiring the use of a wheelchair for mobility. Intervention ingredients were mapped to the six F‐words: functioning, fitness, friends, family, fun, and future. Primary study outcomes were tallied and themed. Evidence strength was assessed using the Australian National Health and Medical Research Council guidelines and the Risk of Bias in Non‐randomised Studies ‐ of Interventions (ROBINS‐I) tool.

**Results:**

Twenty‐four primary research articles were included. Most studies addressed the concept of functioning, fitness, fun, and friends, and few studies addressed family and future. Thirty common intervention ingredients were identified to deliver the F‐words. Eight key outcome areas were identified. Most studies were level IV evidence and possessed a serious level of bias.

**Interpretation:**

Despite identifying numerous intervention ingredients mapped to the F‐words, the overall evidence base remains weak. Strengthening the quality and scope of research is essential to ensure the F‐words framework is applied inclusively for physical activities across diverse populations of children with physical disabilities in community contexts.

## Graphical abstract

This scoping review identifies intervention ingredients that address the F‐words for childhood development in community‐based physical activity programs for children with physical disabilities. An electronic database and hand search of the international literature was conducted. Thirty intervention ingredients were identified to deliver the F‐words, with a range of program outcome areas identified in twenty‐four studies most of which possessed a high risk of bias and were of low‐level evidence.

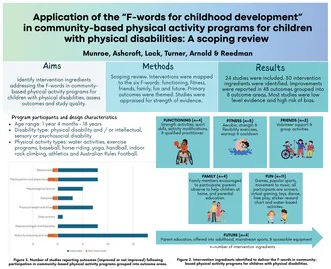

**Abbreviations**
PRISMA: Preferred Reporting Items for Systematic Reviews and Meta‐Analyses
ROBINS‐I: Risk Of Bias In Non‐randomised Studies ‐ of Interventions

**What this paper adds**

- Programmes commonly addressed functioning, fun, fitness, and friends; family and future were less common.
- Outcomes varied widely across psychological, physical, and psychosocial domains.
- Most studies were low‐level evidence, highlighting the need for more rigorous research.

Disability affects the physical activity of children worldwide. Up to 13.5% of the childhood population are reported to have a disability.^1^, ^2^, ^3^, ^4^ Intellectual, psychosocial, and sensory/speech disorders are the most common childhood disabilities.^5^, ^6^ While only approximately 2% of children are reported to have impaired mobility or a physical disability,^3^, ^5^ physical disability in children has a profound impact on their physiological and psychological health.

Children with disabilities are 53% less likely to be physically active compared with typically developing children.^7^ Further, they are more likely to experience health conditions such as asthma, diabetes, anxiety, and depression.^8^, ^9^ More physically active children have higher aerobic and anaerobic fitness, more muscle mass, and less adipose tissue, which decreases the risk of cardiovascular disease when they are older.^10^ Young people who participate in more physical activity experience lower rates of depression, perform better cognitively, and have higher self‐esteem than those who are not physically active.^11^ The World Health Organization recommends children with or without disability aged 5 to 17 years participate in 60 minutes of moderate to vigorous physical activity on a daily basis.^12^

Impairments in body functions and structures associated with a child's physical disability mean they are not physically able to perform tasks like other children.^13^ In some instances, children may attend physical activity programmes, but because of their physical disability they end up observing and not actively participating in the activity.^14^ Inadequate access to buildings and equipment, lack of transport, and absence of inclusive opportunities also make it challenging for children with physical disabilities to attend physical activity programmes.^13^, ^15^ This is further exacerbated by family time constraints and children not feeling supported or included by their peers.^13^, ^16^ More inclusive structured physical activity programmes are needed for children with physical disabilities to overcome such barriers to accessing programmes, increase participation in physical activity, and enhance their health and well‐being.

The F‐words in childhood disability were developed in 2011 to encapsulate the different concepts that should be considered when addressing the health and function of children with disabilities.^17^ The F‐words originally comprised six domains: function, fitness, friends, family, fun, and future.^17^ This was conceptualized on the basis of the World Health Organization's International Classification for Functioning, Disability and Health (ICF) framework developed in 2001 to classify factors that affect health and function of all people (with and without disability).^18^ The F‐words were designed to overcome the ‘medical model’ approach to the ICF framework and translate this into a set of ideas to help meet the needs of children with disability.^17^

More recently, the F‐words have been updated and are now referred to as the ‘F‐words for child development’, recognizing the importance of these concepts for the development of all children, including those with and without a disability.^19^ The concept ‘function’ has been retermed ‘functioning’ to capture the dynamic nature of childhood activity in everyday life, moving away from a static biomedical model.^19^ ‘Freedom’ has also been proposed as an additional F‐word, advocating the importance of individual autonomy and choice.^20^

The F‐words have been used to inform childhood disability programmes internationally, including physical activity and rehabilitation‐based interventions.^21^ Studies have also used the F‐words to evaluate parents' perceptions of modified sports interventions, such as Sports Stars.^22^ Previous literature reviews have synthesized evidence on the application of the F‐words in children with cerebral palsy (CP) aged 0 to 5 years.^23^, ^24^, ^25^ CP encompasses a range of diverse motor and non‐motor features that can result in restricted participation.^26^ However, no previous reviews have examined the application of the F‐words to inform physical activity programmes based in the community or for children with a range of physical disabilities (not limited to CP). Further, no previous reviews have examined the strength of evidence supporting the delivery of the F‐words in community‐based physical activity programmes for children with physical disabilities. The aim of this study was to identify (1) what interventions are used and the intervention ingredients included to address the F‐words for childhood development in community‐based physical activity programmes for children with physical disabilities, and (2) the outcome measures used in the literature and the strength and quality of available evidence.

## METHOD

### Study design

A scoping review was conducted to summarize existing knowledge on how the F‐words have been integrated into community‐based physical activity programmes for children with physical disabilities. The review followed five steps guided by the JBI Manual for Evidence Synthesis:^27^ (1) identifying the research question; (2) identifying relevant studies; (3) study selection; (4) charting of the data; and (5) collating, summarizing, and reporting the results. This scoping review was registered with the Open Science Framework registries (https://doi.org/10.17605/OSF.IO/AHMF6). The Preferred Reporting Items for Systematic Reviews and Meta‐Analyses for Scoping Reviews (PRISMA‐ScR) checklist^28^ guided its preparation. Articles were included that met the population, concept, and context framework criteria.^27^

### The research team

The investigator team was developed in accordance with the ACTIVE framework.^29^ Although designed for systematic reviews, this framework provides a useful guide on how to involve stakeholders in reviews and therefore was used in this project (Table 1). Consequently, the investigator team comprised three doctoral‐trained researchers with clinical experience in childhood disability (i.e. two experienced paediatric physiotherapists and one exercise physiologist). The lead investigator was the primary carer for a child with a rare neurogenetic disorder associated with physical, sensory, and intellectual disability. Involving people with lived experience at the centre of research design reduces the establishment of pre‐conceived ideas and increases the likelihood of results meeting the needs of the end‐user.^30^, ^31^.

**TABLE 1:** Involvement of stakeholder with lived experience described using the ACTIVE framework by Pollock et al.^28^

Framework constructs	Categories	Application in review
Who was involved?	Patients, carers, and/or their families Patients, carers, and/or their families plus other stakeholders Other stakeholders only	Parent of child with a physical disability
How were stakeholders recruited?	Open: fixed/flexible Closed: invitation, existing group or purposive sampling	Purposive sampling: the stakeholder was the initiator of the study
What was the mode of involvement?	Approach? One‐time, continuous, or combined (both one‐time and continuous) Methods? Direct interaction or no direct interaction	Approach: continuous Method: direct interaction via regular teleconferencing
At what stage in the review process did the involvement occur?	Develop question Plan methods Write and publish protocol Develop search Run search Select studies Collect data Assess risk of bias Analyse data Interpret findings Write and publish review Knowledge translation and impact	The stakeholder was involved in all stages of the review process
What was the level of involvement at each stage?	Leading Controlling Influencing Contributing Receiving	The stakeholder was responsible for leading each stage of the review process

### Inclusion criteria

This review included studies that evaluated community‐based physical activity programmes designed for children aged 0 to 18 years with a physical disability (Table 2). For this review, physical disability was defined as impaired gross motor skills (e.g. sitting, walking) ranging from mild limitations in speed, balance, and coordination to severe impairment requiring the use of a wheelchair for mobility (equivalent to levels I–V of the Gross Motor Function Classification System [GMFCS]^32^). Although the primary focus was on children with physical disability, there was a paucity of studies identified that only included participants with a physical disability. Therefore, studies were included if children with intellectual, sensory, or psychosocial disabilities were also represented in the study sample. Community‐based physical activity programmes were considered any planned or structured set of physical activity interventions (any movement requiring energy) delivered in the community setting.^33^ This included both organized and recreational sports activities (structured activities guided by rules, with or without elements of competition)^33^ and structured exercise programmes (planned routine of physical activity with the aim of improving physical fitness).^34^.

**TABLE 2:** Intervention ingredients linked to F‐word concepts adapted from Longo et al.^25^ and Rosenbaum and Gorter.^17^

F‐word	Intervention ingredients
Functioning	Incorporates goal‐directed, task‐oriented training, or self‐initiated movement as part of a functional activity that aims to improve activities of daily living and/or child skill development. Some examples include the following: Sport‐specific skills such as batting, catching, throwing, etc. Strength exercises that use functional movements such as sit‐to‐stands, stair climbing, etc.
Fitness	Incorporates self‐initiated movement, muscle strengthening, or motor control activities that aim to improve body structures and function (such as cardiorespiratory endurance, body composition, muscular strength/power, and flexibility, as well as agility, balance, and co‐ordination). Some examples include the following: Cardiorespiratory exercises such as swimming, running, etc. Strengthening exercises to increase muscle power e.g. weights, resistance bands.
Family	Considers the inclusion of parents, carers, siblings, or extended family members of children with a physical disability. Some examples include the following: Involvement of parents. Involvement of siblings.
Friends	Targets the development of social and emotional connections with others (separate from family members). Some examples include the following: Involvement of other children with/without disability. Involvement of a buddy/peer system.
Fun	Involves child‐directed play, child showing preference, and use of assistive devices to optimize child contentment/enjoyment. Some examples include the following: Involvement of games. Incorporation of music.
Future	Targets the importance of the child's development to prepare them for adulthood, addressing preventive strategies and changing attitudes. Some examples include the following: Opportunity to continue the programme when the child is older. Educational interventions to increase knowledge of child or caregiver.

### Exclusion criteria

Studies were excluded if participants were over the age of 18 years or did not include children with a physical disability. Programmes designed for non‐community‐based contexts (e.g. school or therapy settings) and non‐primary research studies were also excluded.

### Research questions

The research questions were as follows. (1) What community‐based physical activity programmes are available for children with physical disabilities? (2) Which F‐words ingredients compose these intervention programmes? (3) What outcomes are reported for children participating in these programmes? (4) What is the overall strength and quality of the available evidence?

### Identification of relevant studies

An electronic database search of the literature was conducted using databases Cumulative Index of Nursing and Allied Health Literature (CINAHL), Medline, and PsycInfo from inception to September 2025. A hand search of the reference lists of included studies was also conducted. Search terms included: child or children or young person or young people; disability or disabled or disabilities or developmental delay; physical activity or exercise or fitness or physical exercise or sport; and function/functioning or fitness or friends or family or fun or future or F‐words. Only primary research was included. No language restrictions were set. An example of the search strings (CINAHL database) is shown in Appendix S1. Abstracts were initially screened against the inclusion/exclusion criteria by two independent assessors and selected full‐text articles were assessed for eligibility.

### Data charting, appraisal, and synthesis

Data were extracted from eligible studies identified for inclusion, including detail of study characteristics, methods and outcomes, details of the physical activity programme (e.g. type, duration), and how the intervention ingredients delivered in the programme that addressed any of the six F‐words: functioning, fitness, friends, family, fun, and future. An intervention ingredient was defined as a design element of the physical activity intervention which makes the intervention beneficial (e.g. dosage, principles).^25^, ^35^, ^36^, ^37^ In the current review, each intervention ingredient was charted to the F‐word it directly or indirectly addressed. Intervention ingredients could be attributed to multiple F‐words if applicable. Definitions of F‐words were adapted from Longo et al.^25^ and Rosenbaum and Gorter^17^ to make them applicable to the community‐based physical activity context (Table 2). Although freedom has been proposed as an additional F‐word,^20^ it was not assessed as part of this study.

Quality assessments were conducted to assess level of evidence and risk of bias. While scoping reviews do not generally include a critical appraisal of evidence, this was a specific aim of our review which aligns with the methodology outlined in the JBI Manual for Evidence Synthesis.^27^ The critical appraisal was included to inform mapping of evidence strength and not to exclude or rank studies. Quality of evidence was assessed using the Australian National Health and Medical Research Council levels of evidence and grades for recommendations guidelines assigning each study according to its place in the research hierarchy.^38^ Risk of bias was assessed on the basis of methodological quality using the Risk Of Bias In Non‐randomised Studies ‐ of Interventions (ROBINS‐I) tool.^39^

A data dictionary was developed to guide data extraction and piloted by all members of the research team before starting data extraction. See Appendix S2 for a complete copy of the data dictionary. Data extraction was conducted by two independent investigators using a Microsoft Excel spreadsheet. Any disagreements between reviewers were resolved by a third investigator.

### Categorization

Data variables collected during the data charting and synthesis process were analysed using summative content analysis.^40^ This involved coding, counting, and comparing study characteristics, community‐based physical activity programme participants and design, F‐word intervention ingredients, and study outcomes. Study characteristics were grouped according to study type, location, level of evidence, and risk of bias. Participants of physical activity programmes were categorized according to age and disability type. The physical activity programmes were classified according to the activity type, frequency, and duration of the programme. Intervention ingredients described in the physical activity programmes were mapped to the six F‐words using the definitions outlined in Table 2. If an intervention ingredient was applicable to more than one F‐word, it was assigned to all relevant F‐word concepts in accordance with existing literature.^22^, ^23^ For example, an aerobic activity such as soccer would be assigned to functioning (as it targets sports skills, which are important in childhood development), fitness (as it increases cardiorespiratory function), friends (as it promotes social connections through working as a team), and fun (as soccer is an enjoyable sport). Primary study outcomes reported to examine the effect of participation in the physical activity programme were tallied and themed using inductive content analysis.^41^ Data categorization and presentation of themes were completed through team discussion.

## RESULTS

### Study selection

A total of 2011 abstracts (after removal of duplicates) were screened against the inclusion/exclusion criteria. Sixty full‐text articles were reviewed for eligibility, including three non‐English language papers transcribed into English. Of the 60 full‐text articles reviewed, a total of 24 articles were included and critically appraised in the review. See Figure S1 for the PRISMA flowsheet.

### Study characteristics

The highest proportion of studies were published in the USA (46%, *n* = 11), with other studies originating from Australia,^42^, ^43^ Brazil,^44^, ^45^, ^46^, ^47^ Ghana,^48^ Canada,^47^ Denmark,^49^ Serbia,^50^ South Africa,^51^ Spain,^52^ and Turkey.^53^ Studies were published between 2002 and 2025. Most study participants were children with physical disabilities who participated in the physical activity programme (*n* = 19), four studies included the parent and/or carer of child participants (*n* = 4), and one study included both. Sample sizes ranged from 3 to 91 participants. A summary of studies included in the review is presented in Table S1.

Most studies were non‐randomized intervention studies, with 46% (*n* = 11) using a pre–post design. There were three randomized control trials.^43^, ^46^, ^47^ Other study designs included case series,^52^, ^54^, ^55^ single group interrupted time series,^49^, ^56^ causal‐comparative design,^53^, ^57^ mixed‐methods,^58^ retrospective cohort,^59^ and qualitative descriptive design.^45^ Most studies were level IV evidence (71%, *n* = 17); however there were four studies classified as level III evidence,^60^, ^61^, ^62^ and three studies as level II.^43^, ^46^, ^47^ When assessed for risk of bias using the ROBINS‐I tool,^39^ most studies were identified as possessing a serious level of bias (58%, *n* = 14), six possessed a moderate level of bias,^43^, ^45^, ^46^, ^49^, ^50^ three studies possessed a critical level,^44^, ^51^, ^52^ and one a low level^47^ (Table S1). See Table S2 to review the complete critical appraisal of study bias.

### Programme participants and design features

Programme participants were aged from 1 year 4 months to 18 years of age. All 24 studies included participants who lived with a physical disability. Fourteen studies comprised programme participants all reported to live with a physical disability,^43^, ^44^, ^45^, ^46^, ^47^, ^48^, ^50^, ^51^, ^52^, ^53^, ^56^, ^63^, ^64^, ^65^ with three studies including participants who lived with a co‐existing intellectual, sensory, and/or psychosocial disability.^63^, ^64^, ^66^ Ten studies included programme participants with a mixture of physical, intellectual, or sensory disability.^42^, ^49^, ^55^, ^57^, ^58^, ^59^, ^66^, ^67^, ^68^, ^69^ The most common diagnosis reported was CP (*n* = 16) with GMFCS levels ranging from I to V. A summary of programme participants is presented in Table S1.

Physical activity programmes ranged from water activities such as aquatics, water sports, surfing, and/or swimming;^44^, ^50^, ^51^, ^52^, ^57^, ^58^, ^66^, ^67^, ^69^ exercise programmes such as aerobics, strength, and/or fitness classes;^48^, ^54^, ^68^, ^70^ golf;^53^ soccer;^43^, ^44^, ^45^, ^46^, ^47^ baseball;^43^, ^46^, ^47^, ^59^ horseback riding;^45^, ^65^ yoga;^55^ handball;^43^, ^45^, ^46^, ^47^ athletics;^45^ indoor rock climbing;^49^ gymnastics;^56^ and Australian Rules Football (Auskick).^42^ Most physical activity sessions were 60 minutes in duration. Session frequency ranged from a one‐off session to one to four sessions per week, most lasting 8 to12 weeks with one programme lasting up to 20 months (Table S1). Nineteen of the 24 programmes were conducted in a small group, with the remaining five interventions conducted one‐on‐one with the intervention provider (e.g. volunteer, parent, physical therapist).

### F‐word intervention ingredients

Most studies described physical activity programmes that included interventions to address functioning (92%, *n* = 22), fun (92%, *n* = 22), fitness (88%, *n* = 21), and friends (84%, *n* = 20). Only a small number of programmes described intervention ingredients that addressed family (42%, *n* = 10) or future (29%, *n* = 7). A total of 30 intervention ingredients were identified from the literature aligned with the F‐words. An overview of the intervention ingredients identified in the community‐based physical activity programmes mapped to the six F‐words is presented in Figure 1.

**FIGURE 1:**
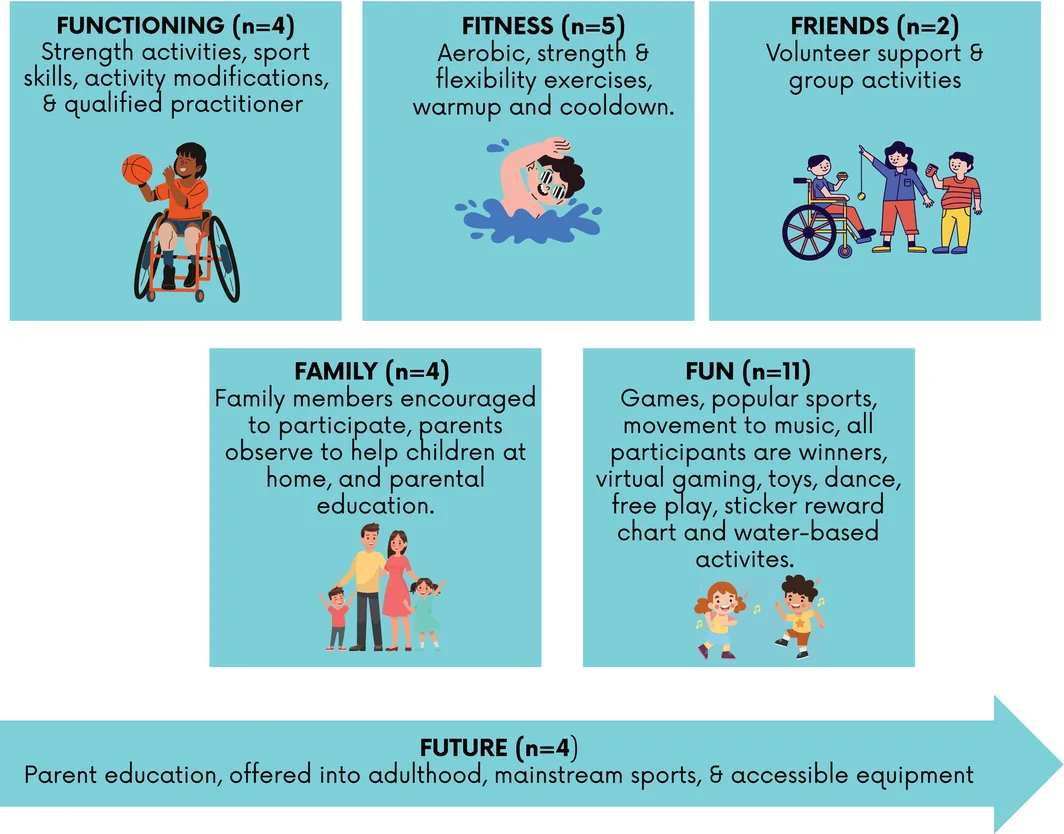
Intervention ingredients identified to deliver the F‐words in community‐based physical activity programmes for children with physical disabilities. *n* = number of intervention ingredients.

### Functioning

Four common intervention ingredients featured in physical activity programmes across the 22 studies that addressed the concept of functioning. Sports skills (e.g. batting, bowling, surfing, golf) were most common (79%, *n* = 19), followed by strength activities using functional movements (e.g. marching, skipping, running) (54%, *n* = 13). Seven studies (29%) used activity modifications to address physical activity (sports field made wheelchair accessible, difficulty of activity tailored to individual capability), and nine studies (38%) reported support of a qualified practitioner.

### Fitness

Of the 21 studies of physical activity programmes that addressed the concept of fitness, five common intervention ingredients were identified. The most common intervention ingredients were strength exercises (yoga, sports skills, balance seated on horse) (71%, *n* = 17) and aerobic exercises (swimming, running) (54%, *n* = 13). Six (25%) studies also featured flexibility exercises (e.g. stretching), as well as warm‐up (38%, *n* = 9) and cool‐down exercises (33%, *n* = 8).

### Friends

While the concept of friends was addressed in 20 studies, there were only two types of intervention ingredient identified. This included use of group activities (79%, *n* = 19) and volunteers to support participants (33%, *n* = 8). This allowed for the development of friendships with other children with disabilities and people in the community.

### Family

Four intervention ingredients were identified to address the concept of family across 10 studies. The most common intervention ingredients identified were encouragement provided to families to participate in the programme (25%, *n* = 6) and the provision of education to parents on how to care for the child, for example nutrition, exercises, and wellness (17%, *n* = 4). One study described how family members were included in the volunteer structure and another how parents were provided with opportunities to interact with each other.

### Fun

Fun was presented in a range of ways in physical activity programmes, with 11 intervention ingredients described across the 22 studies. Game‐based interventions were most common (63%, *n* = 15), followed by the use of popular sports (46%, *n* = 11), movement to music (13%, *n* = 3), and water‐based activities (13%, *n* = 3). Other less frequently reported intervention ingredients identified included free play, obstacle courses, the use of toys, the use of reward charts or award systems, virtual gaming systems, and all participants are winners.

### Future

Future was addressed through four intervention ingredients from seven different community‐based physical activity programmes. Parent education was the most reported intervention ingredient addressing future, with four studies (17%) focusing on educating parents on sports programmes and safety outside the programme. Additional intervention ingredients identified in single studies encompassed offering the programme to older children, integration of participants into mainstream sports, and the provision of equipment made available to participants outside the programme.

### Participation outcomes

Across the 24 included studies, 48 different outcome types were reported resulting from participation in community‐based physical activity programmes. The 48 different outcome types identified were categorized into eight common outcome areas: daily activities; physical health and fitness; muscle strength and flexibility; motor functioning and mobility; symptoms; psychological factors; participation and physical activity; and relationships (Figure 2). All outcome areas were reported to improve following participation in the programme. A few studies also reported no improvement in the following five outcomes: physical health and fitness; motor functioning and mobility; symptoms; participation and physical activity; and relationships. No adverse events or negative outcomes were reported in any of the studies.

**FIGURE 2:**
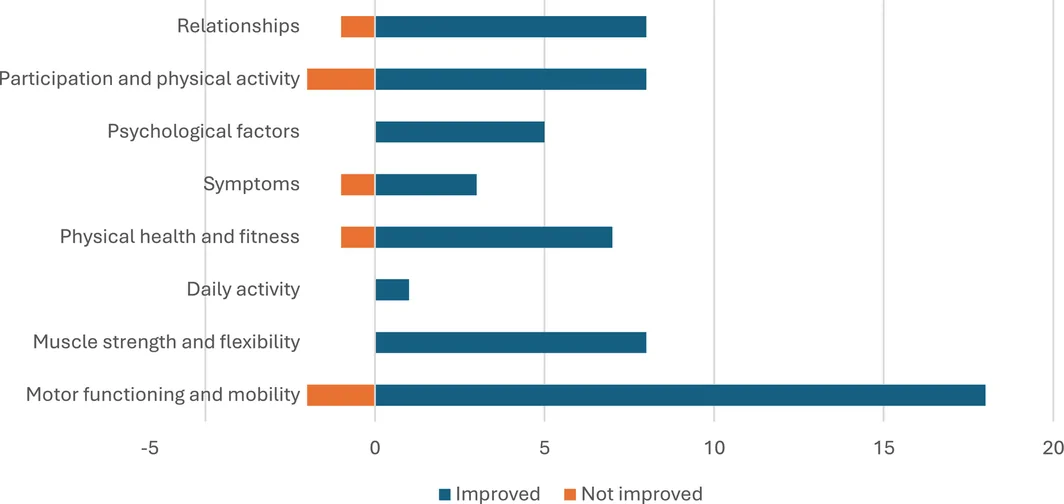
Number of studies reporting outcomes (improved or not improved) following participation in community‐based physical activity programmes grouped into outcome areas.

Improvements in daily activities were noted in areas such as sleep and feeding. Gains in physical health and fitness included enhanced cardiorespiratory fitness, increased fat‐free mass, and improved bone mineral density. Participation in these programmes was also associated with increases in muscle strength and flexibility, specifically grip and core strength, as well as muscle endurance and flexibility. Enhancements in motor functioning and mobility were frequently reported, encompassing walking speed, energy expenditure, and walking distance, as well as gross motor and functional skills such as transfers. Studies also described improvements in upper limb function, independence, and global ability.

Several symptom‐related outcomes demonstrated positive change, including reductions in pain, spasticity, and seizures, along with improved anal sphincter control. Psychological benefits were also reported, particularly in relation to mood, attention, and self‐confidence. In terms of participation and physical activity, findings indicated greater knowledge of the importance of physical activity and increased engagement across home, school, and community settings. Finally, improvements in relationships were highlighted, with participants reporting stronger connections both with family members and with peers.

## DISCUSSION

To our knowledge, this is the first review to explore how to address the F‐words in community‐based physical activity programmes for children with physical disabilities. A wide range of physical activity types were reported in the literature, integrating a variety of intervention ingredients to address the F‐words for childhood development. Intervention ingredients addressing functioning, fitness, friends, and fun were the most common, with only a small number of studies identified that addressed the concept of family or future. All included studies reported positive outcomes following participation in community‐based physical activity programmes, although there were some outcome areas that were reported to not improve in a small number of studies. Further, the overall body of evidence was limited by low methodological quality and a high risk of bias.

The community‐based physical activity programmes identified in this review encompassed a range of modified sport programmes including water‐based activities, athletics, horseback riding, indoor rock‐climbing, gymnastics, and sport‐based activities. Modified sport programmes aim to improve fundamental motor skills and promote lifelong participation in physical activity by modifying the rules and equipment of traditional sports.^71^, ^72^, ^73^, ^74^ Early evidence has suggested modified sport interventions are useful to improve gross motor function in children with CP,^75^ and fundamental motor skills in children with autism spectrum disorder.^76^ However, the intervention ingredients required for a successful modified sport intervention for children with physical disabilities are lacking.^60^ As such, little is known about how to successfully implement modified sport interventions for children with physical disabilities other than CP in a community‐based setting or how to address the F‐words for childhood development.

Intervention ingredients most frequently featured in physical activity programmes addressed the concept of functioning or fitness which commonly emerged together. It is subsequently not surprising that the most frequently reported positive outcome areas (motor functioning and mobility, and physical health and fitness) are closely aligned to functioning and fitness. Leite et al.^23^ similarly reported functioning (previously termed function) and fitness to be the most common F‐words to be integrated into non‐invasive interventions for ambulant children with CP. Merging of functioning and fitness was also noted in a study that asked parents to report their experiences of modified sports interventions.^45^ The confluent nature of functioning and fitness is probably due to children needing fitness to perform functional activities. For example, one needs to have cardiorespiratory fitness to run, skip, and engage in play, which is a functional activity.

Activity modifications featured in some physical activity programmes evaluated in our review to address the child's disability and optimize participation. Adjusting the activity type or difficulty to match a child's functional and fitness level is a key strategy in modified sports interventions.^77^ Another strategy to support the child's function was the support of qualified practitioners such as physical therapists. The integration of a qualified practitioner is also a common feature of modified sports interventions.^78^ The support of qualified practitioners brings expertise in programme design to ensure the activities increase functional mobility and activity levels, reduce impairments of body structure and function, and optimize participation.^79^

While the focus of this review was on children with physical disabilities, it is important to note that functioning not only encompasses musculoskeletal or movement‐related functions but also intellectual, psychosocial, and sensory functions.^77^ This is particularly important in children with disabilities, as many live with multiple disabilities, not limited to physical impairments.^80^ Use of strategies such as verbal, musical, visual, tactile reinforcement/prompts, as well as modelling behaviours should also be considered when designing modified physical activity programmes for children with disabilities to meet needs beyond just the physical.^77^

Positive outcomes were reported in participants' relationships both with family members and with peers. As most programmes were delivered in a group setting, we identified several physical activity programmes that addressed the F‐word concept of friends. However, there were significantly fewer explicit intervention ingredients identified that considered the concept of family. In contrast, previous reviews on interventions for children with disabilities in primarily therapy settings reported the concept of family to be more readily addressed than friends.^23^, ^81^ The objective of therapy‐based interventions differs from community‐based programmes because they are usually one–one–one and goal‐driven, aimed at training the parents/carers, optimizing function, and minimizing impact of their disability.^82^ Alternatively, the goal of community‐based programmes for people with a disability is to optimize participation, social interaction, and engagement, promoting equal opportunities.^83^ Children therefore do not have the same opportunity to form social connections in therapy compared with community‐based settings. However, family is an integral component of both therapy and community‐based activities.

It is important that family is considered when designing physical activity programmes for children with disabilities, as parents or carers experience challenges that can often lead to emotional and physical fatigue, stress and anxiety, as well as social isolation.^84^ Family members, such as parents and siblings, were integrated into programmes identified in this review, by offering opportunities for their participation and interaction between parents/carers. Parents and carers of children with disability have reported that informal supports from community groups help reduce negative emotions and increase resilience.^85^ However, there were no specific intervention ingredients identified in this review that focused on the well‐being of parents or carers.

Several studies in this review described educational interventions to support the parent care for their child, such as education on the exercise programme and nutrition. Parents and carers have a direct influence on the psychological and social development of young children in physical activity.^86^ A lack of parental awareness of the benefits of physical activity has been reported to affect parental support of children with physical disability, and subsequent involvement in physical activity.^87^ Educational interventions for parents or carers may assist to overcome such barriers, empowering parents and carers with knowledge and skill to support their children to be physically active, in the short and long term, which also encapsulates the concept of future.

No programmes were identified to include strategies to support siblings of children with disability in the literature review. Siblings of a child with a disability grow up with considerable stress, reporting complex feelings of isolation, resentment, guilt, anger, and embarrassment negatively affecting their mental health and social development.^88^, ^89^ Integrating opportunities for siblings in sporting activities for children with disability can provide siblings opportunities to share experiences and offer support to other family members that understand the challenges of having a family member with a disability.^84^, ^90^

Several studies included in the review reported children were more likely to participate in physical activity following participation in community‐based physical activity programmes. Motivation plays a critical role in children participating in physical activity particularly as they get older and motivation often decreases.^15^ Our review found most programmes featured activities that are perceived as fun and motivate children to participate, in particular the use of play through games, toys, and sports, as well as music. There is extensive research on the functional benefits play has on the cognitive, social, and physical health and development of children.^91^ Music has also been reported to help children with disabilities communicate^92^ as well as generate fun and enjoyment.^93^ Understanding what motivates the children and the development of tailored strategies to optimize fun is integral to achieving active participation in physical activity programmes.

Several studies reported improvements in participants' disability‐related symptoms following participation in physical activity programmes, along with enhancements in physical health and fitness, both of which are important for long‐term quality of life. Yet, future was the least considered F‐word in physical activity programmes identified in the review. This aligns with findings from previous reviews which also reported a lack of intervention ingredients to address the concept of future.^23^, ^24^ This may be attributed to the limited availability of physical activities available in the community for children with physical disabilities, including participation in mainstream sports.

Generating opportunities for children with physical disabilities to participate in mainstream sport promotes community participation and engagement. However, not all children with disabilities can participate in mainstream sports. As discussed by Clutterbuck et al.,^78^ mainstream sports are not designed for people with disabilities, rather they are designed for people with age‐appropriate sports skills. While mainstream sports are popular and therefore readily available, people with disabilities frequently experience barriers that prevent them from participating.^78^ In contrast, modified sporting interventions overcome many of these barriers, enabling people with physical disabilities and/or intellectual disabilities to participate. However, opportunities to participate in modified physical activity programmes are limited, particularly in regional and rural areas.

Our critical appraisal of the research identified revealed a high risk of bias and low level of evidence. While previous reviews have not critically appraised the evidence, it was noted that one previous scoping review conducted by Leite et al.,^23^ on the application of F‐words in young ambulant children with CP, primarily included randomized control trials. Randomized control trials are the highest level of primary research (National Health and Medical Research Council levels of II). However, our review included mostly non‐randomized studies with predominantly level III and IV evidence. Several studies were limited to people with CP and less severe physical disabilities.

CP is a permanent non‐progressive neurological disorder that effects movement and posture usually caused by injury to the foetal or infant brain.^94^ Existing reviews identified also focused on the application of the F‐words in children with CP.^23^, ^24^, ^25^ This is probably attributed to CP being the most common childhood disability associated with physical impairment.^95^ While many childhood neurological disorders and syndromes share similar characteristics to CP, there are distinct differences.^96^ Generalizing results about children with other diagnosis has the potential to disadvantage these children and result in the child's needs not being met. The feasibility of conducting studies of high‐level evidence in such rare conditions or heterogeneous populations, however, can be challenging owing to feasibility of recruiting adequate sample sizes because of the rarity of some neurodevelopmental disorders. Future studies should consider grouping children on the basis of similar levels of function and characteristics common across different diagnosis.

This paper has several strengths and some limitations. The data extraction process followed in the scoping review was extremely rigorous, with two independent reviewers responsible for data extraction and a third reviewer used to resolve any discrepancies reduced the introduction of bias. The inclusion of a critical appraisal of the evidence has not been previously reported in published reviews mapping the F‐words constructs. The use of the National Health and Medical Research Council guidelines^38^ and ROBINS‐I tool^39^ also provided a validated approach to assessing the quality of studies extracted. The literature review was, however, restricted to published research only and did not include grey literature, which may have excluded some physical activity programmes. Despite the development of a data dictionary to ensure a clear definition of each F‐word was developed at the start of this study, a key challenge of this review was the inconsistent reporting of intervention ingredients, which made it difficult to categorize ingredients according to the F‐words. Future research may be improved by clearly defining and describing the intervention ingredients, as demonstrated in a few studies included in this review that more clearly described intervention ingredients.^46^, ^58^, ^69^, ^70^ Use of the ‘F‐words Lens Tool’^97^ may further support researchers and clinicians in designing community‐based physical activity interventions for children with physical disabilities that intentionally address all six F‐words.

## CONCLUSION

This scoping review found a range of community‐based physical activity programmes for children with physical disabilities, from modified sports to structured exercise interventions. Most programmes addressed the F‐words of functioning, fitness, friends, and fun, with limited attention given to family and future. Participation was consistently associated with positive outcomes, including enhanced physical and psychological health, relationships, and well‐being. Despite these encouraging findings, the evidence base was limited, with most studies classified as low quality and at high risk of bias. Research also largely focused on children with CP, with minimal inclusion of those with co‐existing intellectual, psychosocial, or sensory disabilities. To strengthen the evidence and guide practice, future studies should adopt rigorous designs and broaden their scope to ensure the F‐words framework is applied inclusively across diverse populations of children with physical disabilities in community contexts.

## FUNDING

No funding was received for the preparation of this manuscript.

## CONFLICT OF INTEREST STATEMENT

The authors have stated that they had no interests that might be perceived as posing a conflict or bias.

## Supporting information


**Appendix S1.** Search strategy conducted September 2025

**Appendix S2.** Data dictionary.

**Figure S1.** PRISMA Flowsheet depicting the systematic method followed to identify and screen articles.

**Table S1.** A summary of study characteristics and community‐based physical activity program design features included in review

**Table S2.** Critical appraisal of study bias.

## Data Availability

Data sharing not applicable ‐ no new data generated.
