# Supplementary material for: The F‐words for childhood development in community‐based physical activity programmes for children with physical disabilities: A scoping review
Source: Dev Med Child Neurol. 2026 May 11;68(11):1501–13. doi: 10.1111/dmcn.70306 (PMC13649294; doi:10.1111/dmcn.70306)
Supplement: Supplementary file 1 [file DMCN-68-1501-s005.pdf]

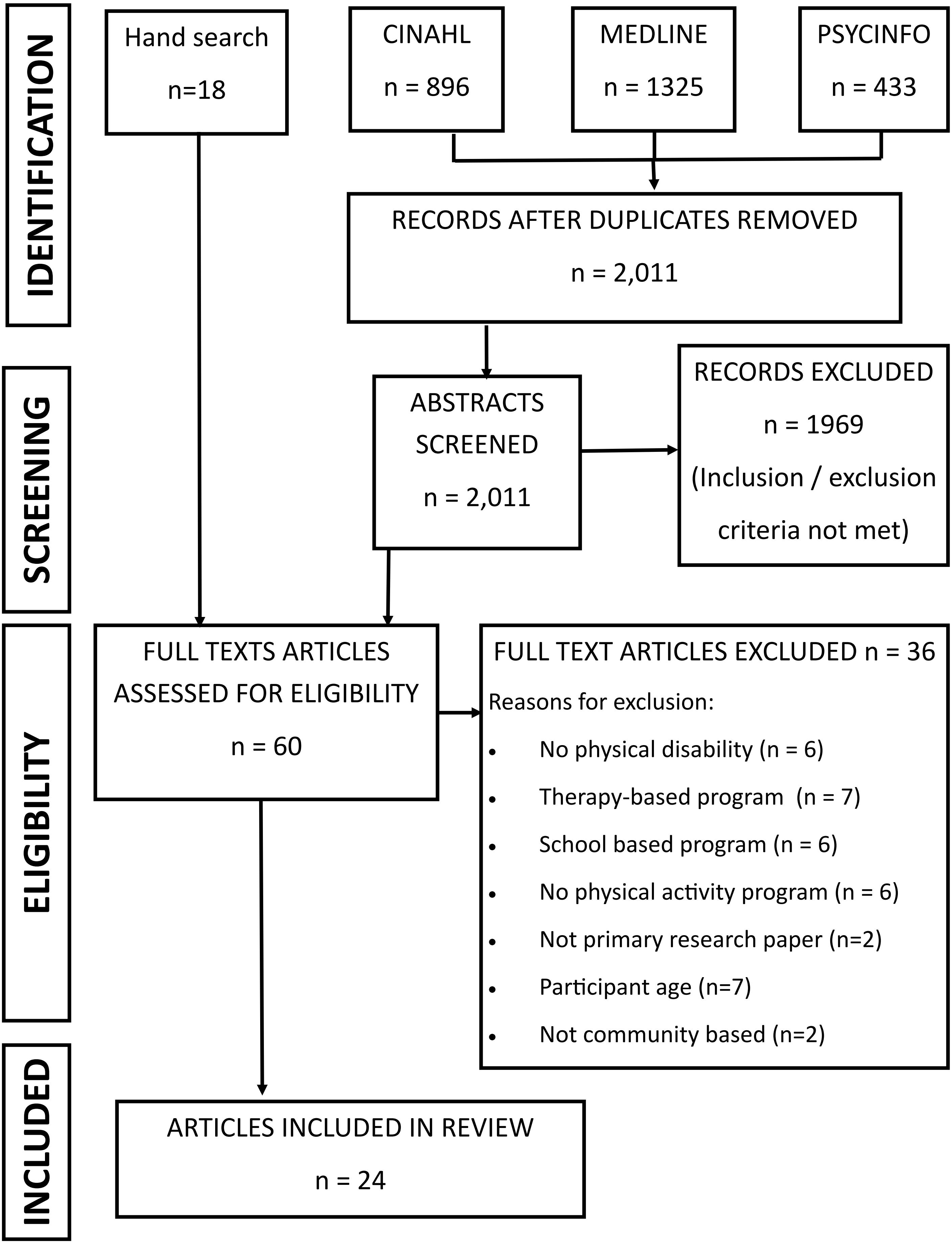

Figure S1 PRISMA Flowsheet depicting the systematic method followed to identify and screen articles
