# Supplementary material for: The F‐words for childhood development in community‐based physical activity programmes for children with physical disabilities: A scoping review
Source: Dev Med Child Neurol. 2026 May 11;68(11):1501–13. doi: 10.1111/dmcn.70306 (PMC13649294; doi:10.1111/dmcn.70306)
Supplement: Supplementary file 2 [file DMCN-68-1501-s002.docx]

| **Item to be extracted** | **Definition** |
| --- | --- |
| **Year** | Year of publication (usually found on the first page of the PDF, usually in the top or bottom corner). |
| **Country** | May be included in the methods section. If not, look at the affiliations of the authors listed on the first page of the PDF. |
| **Study design** | Usually listed in the first few lines of the methods section. Examples may include:   - Exploratory study. - Mixed methods study. - Pre-post intervention study. - Case report/series. |
| **Sample** | Number of participants and category (e.g., participants, parents, providers of the program). |
| **Physical activity (type and frequency/ duration)** | Details of the structure of the physical activity program:   - Type = what is the intervention? (e.g., adaptive sport, skill training). - Frequency = how often is the program running? (e.g., weekly, monthly). - Duration = how long does the program run for? (e.g., number of weeks or months). |
| **Age (users of program)** | Age of the participants who completed the physical activity program. May be listed as an age range or mean (with standard deviation). |
| **Disability (participants)** | Disability of the participants who completed the program. If multiple disabilities are reported, please include the percentage/number of each. |
| **Study methods** | How did the researchers identify the effects of the program? Examples may include:   - Survey. - Experimental study (assessments usually at baseline and after the program). |
| **Functioning** | Describe the component(s) of the program that incorporates goal-directed, task orientated training, or self-initiated movement as part of a functional activity that aims to improve activities of daily living and/or child skill development.  Some examples include:   - Sport-specific skills such as batting, catching, throwing, etc. - Strength exercises that use functional movements such as sit-to-stands, stair climbing, etc. |
| **Fitness** | Describe the component(s) of the program that Incorporates self-initiated movement, muscle strengthening or motor control activities that aim to improve body structures and function (such as cardiorespiratory endurance, body composition, muscular strength/power, and flexibility, as well as agility, balance, and co-ordination).  Some examples include:   - Cardiorespiratory exercises such as swimming, running, etc. - Strengthening exercises to increase muscle power e.g. weights, resistance bands. |
| **Family** | Describe the component(s) of the program that considers the inclusion of parents, carers, siblings, or extended family members of children with a physical disability.  Some examples include:   - Involvement of parents. - Involvement of siblings. |
| **Friends** | Describe the component(s) of the program that targets the development of social and emotional connections with others (separate from family members).  Some examples include:   - Involvement of other children with/without disability. - Involvement of a buddy/peer system. |
| **Fun** | Describe the component(s) of the program that involves child-directed play, child showing preference, and use of assistive devices to optimise child contentment / enjoyment.  Some examples include:   - Involvement of games. - Incorporation of music. |
| **Future** | Describe the component(s) of the program targets the importance of the child’s development to prepare them for adulthood, addressing preventative strategies and changing attitudes.  Some examples include:   - Opportunity to continue the program when the child is older. - Educational interventions to increase knowledge of child or care giver. |
| **Study outcomes** | What were the key outcomes of the study?  Consider the primary outcomes and what is important.  List as much detail as you think is relevant. |
| **Level of evidence** | State level of evidence I-V guided by the NHMRC levels of evidence and grades for recommendations  for developers of guidelines. |
| **Risk of bias** | State risk of bias per ROBINS-I tool assessment findings. |
