# Supplementary material for: The F‐words for childhood development in community‐based physical activity programmes for children with physical disabilities: A scoping review
Source: Dev Med Child Neurol. 2026 May 11;68(11):1501–13. doi: 10.1111/dmcn.70306 (PMC13649294; doi:10.1111/dmcn.70306)
Supplement: Supplementary file 3 [file DMCN-68-1501-s003.docx]

**Appendix 1.** Search strategy conducted September 2025

**Main keywords used in the databases**

child

AND

disability OR disabilities OR disabled

AND

exercise OR fitness OR physical activity OR physical exercise OR sport

AND

function OR fitness OR friends OR family OR fun OR future OR F-words

**CINAHL**

STRATEGY

Search:  **( MH( child ) AND ( disability or disabilities or disabled ) AND ( exercise or "physical activity" or "physical exercise" or sport ) AND ( function or fitness or friends or family or fun or future or "F-words" ) )**

Expanders: **Apply** **equivalent subjects**

Search modes: **Find all my search terms**
