# Supplementary material for: The F‐words for childhood development in community‐based physical activity programmes for children with physical disabilities: A scoping review
Source: Dev Med Child Neurol. 2026 May 11;68(11):1501–13. doi: 10.1111/dmcn.70306 (PMC13649294; doi:10.1111/dmcn.70306)
Supplement: Supplementary file 4 [file DMCN-68-1501-s004.docx]

Table S1: A summary of study characteristics and community-based physical activity program design features included in review

| **Source** | **Study design** | **Location** | **Methods** | **Sample** | **Sample size** | **Level of evidence*** | **Risk of Bias**** | **Diagnosis** | **Participant Age** | **Physical activity** | **Session frequency (per week)** | **Session duration (per session)** | **Session duration (weeks)** |
| --- | --- | --- | --- | --- | --- | --- | --- | --- | --- | --- | --- | --- | --- |
| Ansa et al., 2021 | Pre-post study design | Ghana | Gross motor function, walking distance in 30 seconds and quality of life of children and parents tested before and after the program. | Program participant | 10 | IV | Serious | Spastic CP  (GMFCS I-II) | 12-16 years (range)  Mean not reported. | Aerobic exercise program | 4 sessions | 50 minutes | 8 weeks |
| Armitano-Lago et al., 2015 | Pre-post study design | USA | Cardiorespiratory endurance, flexibility, muscular strength and muscle endurance tested before and after the program. | Program participant | 16 | IV | Serious | Autism, Down Syndrome, Autism-nonverbal, Global Developmental Delay, ADHD, Microcephaly, very low muscle tone, learning difficulties, sensory integration disorder, hyperkinetic, Dandy walker syndrome, Tourette Syndrome | 5-15 years (range)  10.81 ± 3.38 (mean ± SD) | Surfing | 2 sessions | 60 minutes | 8 weeks |
| Ballington & Naidoo, 2018 | Pre-test post-test control study design | South Africa | Gross motor function was measured before the program, after the program and after the cross-over period. | Program participant | 10 | III-3 | Critical | CP (GMFCS I-III) | 8-12 years (range)  11 ± 0.008 years (mean ± SD) | Swimming (Halliwick Concept) | 2 sessions | 30 minutes | 8 weeks |
| Christensen et al., 2017 | Single group interrupted time series | Denmark | Physiological, psychological and cognitive tests were conducted two times before and one time after the program. | Program participant | 17 | IV | Moderate | CP (GMFCS I-II) | 11-13 years (range)  With CP: 11.6 ± 0.8 years (mean ± SD)  Without CP: 11.8 ± 0.9 years (mean ± SD) | Indoor climbing | 3 sessions | 150 minutes | 3 weeks |
| Clapham et al., 2020 | Causal-comparative research design | USA | Fitness, core muscle strength, muscle endurance, flexibility, fat-free mass and bone mineral density measured before and after the programs. | Program participant | 91 | III-2 | Serious | Intellectual and learning disabilities, down syndrome, several autism spectrum disorders, microcephaly, global developmental delays, dandywalker syndrome, heart defects, and hypothyroidism. | 5-18 years (range)  Surfing group: 12.55 ± 3.83 years (mean ± SD)  Pool group: 11.1 ± 4.55 years (mean ± SD) | Surfing / Pool play | 2 sessions | 60 minutes | 8 weeks |
| Clutterbuck et al., 2022 | Randomised controlled trial | Australia | Gross motor function, physical activity competence and quality of life were measured before the program, after the program and after the follow up period. | Program participant | 54 | II | Moderate | CP (GMFCS I-II) | 6-12 years (range)  Sport Stars group: 8.94 ± 2.05 years (mean ± SD)  Control group: 8.64 ± 2.04 years (mean ± SD) | Sport Stars program (soccer, handball, basketball and athletics) | 1 session | 60 minutes | 8 weeks |
| Cook et al., 2015 | Single group interrupted times series | Canada | Habitual physical activity, physical self-perception and gymnastics skills were measured before the study, after Phase 1 (intervention or control) and after Phase 2 (crossover period). | Program participant | 5 | IV | Serious | CP (GMFCS I-III) | Range not reported.  9.8 ± 1.3 years | Gymnastics class | 2 sessions | 60 minutes | 6 weeks |
| Escobar Torres et al., 2019 | Case series | Spain | Parental qualitative surveys at the end of the first and second years to gather information on symptoms. Functional performance measures, anthropometric measures, heart rate, and joint mobility of the children tested at before and after each year. | Parent / carer | 3 | IV | Critical | Stage III Rett syndrome | 4, 6 and 7 years (range)  Mean not reported. | WaterFit MITAF (aquatic program) | 3 sessions | 30 minutes | 20 months |
| Feitosa et al., 2017 | Pre-post prospective study | Brazil | Quality of life, evaluation of intervention results, and biopsychosocial profile of the child was measured before the program and after 1-year. | Program participant | 17 | IV | Critical | CP (GMFCS I -II) | 7-14 years (range)  10.6 ± 1.7 years (mean ± SD) | Soccer, swimming or both soccer and swimming | Soccer (1 session), Swimming (2 sessions), both (3 sessions) | Not reported | 12 months |
| Fragala-Pinkham et al., 2006 | Pre-test post-test design | USA | Isometric lower limb muscle strength, walking energy expenditure, functional mobility, and fitness were measured before and after the program. | Program participant | 28 | IV | Serious | CP (GMFCS I-III), Duchenne muscular dystrophy, traumatic brain injury, and gross motor delay | 6-14 years (range)  9.1 ± 2.3 years (mean ± SD) | Community based exercise program | 2 sessions | 60 minutes | 16 weeks |
| Fragala-Pinkham et al., 2005 | Case series | USA | Strength, shuttle run, and gross motor skills measured before the program, after the group exercise program, and after the home exercise program. | Program participant | 9 | IV | Serious | CP (GMFCS I -III), developmental disorder / delay, anoxic brain injury, developmental coordination disorder | 5-9 years (range)  7.68 ± 1.27 years (mean ± SD) | Community and home based aerobic and strength exercise program | 2 sessions | 60-70 minutes | Community program (14 weeks), Home program (12 weeks) |
| Fragala-Pinkham et al., 2008 | Pre-post study design | USA | Strength, motor skills and fitness were assessed | Program participant | 16 | IV | Moderate | CP (GMFCS I-II), developmental delay, myelomeningocele, Down syndrome, nonverbal learning disorder, autism, otopalatodigital syndrome, pervasive developmental delay not otherwise stated | 6-11 years (range)  9 years 7 months ± 1 year 4 months (mean ± SD) | Aquatic exercise program | 2 sessions | 50 minutes | 14 weeks |
| Fragala-Pinkham et al., 2010 | Pre-Post Design | USA | Swimming skills and parent-reported physical activity of the child were assessed before the program, after the program and after follow up. | Program participant | 16 | IV | Serious | CP, autism spectrum disorder, Down syndrome, Myelomeningocele, developmental delay, nonverbal learning disorder, otopalataldigital syndrom | 6-12 years (range)  9 years 7 months (mean) | Aquatic exercise program | 2 sessions | 45 minutes | 14 weeks |
| Gercek et al., 2022 | Causal-comparative research design | Türkiye | Measures of gross motor function, muscle spasticity, muscle strength, walking endurance, flexibility, and balance completed before and after the 12-week program. | Program participant | 19 | III-2 | Serious | Spastic unilateral CP (GMFCS levels I-II) | 6-12 years (range)  Virtual Golf group: 8.22 ± 1.71 years (mean ± SD)  Traditional Golf group: 8.50 ± 2.50 years (mean ± SD) | Golf (traditional or virtual) | 3 sessions | 60 minutes | 12 weeks |
| Haley et al., 2006 | Pre-post study design | USA | Functional mobility measured before and after the program. | Program participant | 28 | IV | Serious | Neuromuscular disorders (e.g. CP, traumatic brain injury, Duchenne muscular dystrophy), developmental disabilities (e.g. Williams syndrome, Soto syndrome, and DeGeorge syndrome), and medical conditions (congenital heart disease, lupus) | 6-14 years (range)  9.1 ± 2.32 years (mean ± SD) | Group exercise program | 2 sessions | 60 minutes | 16 weeks |
| Howells et al., 2019 | Pre-post study design | Australia | Online mixed-methods surveys completed by parents before each ‘Pre-Learn’ day, within 4-weeks after the event, and after the conclusion of the official Auskick season (approximately 18-weeks after the second survey). | Parent / carer | 15 | IV | Serious | ASD, CP, Down syndrome | 5-11 years (range)  8.16 ± 2.23 (mean ± SD) | AUSKICK (Australian rules football program) | Not reported | 60 minutes | Total of two (2) "Pre-Learn Days" |
| Jorgić et al., 2024 | Pre-post study design | Serbia | The study included a 12-week control period (no intervention) followed by a 12-week intervention period (12-week aquatic program). Gross motor function, water orientation, walking endurance, walking speed, and energy expenditure measured before the control period, after the control period, and after the program. | Program participant | 18 | IV | Moderate | CP (GMFCS I-III) | Range not reported.  12.3 ± 3 years (mean ± SD) | Pool based exercise program | 3 sessions | 60 minutes | 12 weeks |
| Lyons et al., 2009 | Retrospective cohort study | USA | Mixed-methods survey of parents to determine perceptions of the program. | Parent / carer | 42 | IV | Serious | Cognitive, physical, and/or sensory disabilities | 4-17 years (range)  Mean not reported. | Charleston Miracle League (adaptive community baseball program) | 1 session | NA | NA |
| Price et al., 2022 | Mixed methods design | USA | Semi-structured interviews with parents conducted before and after the program. Intervention Rating Profile-15 (IRP-15) measured after the programs to objectively measure social validity following completion of each portion of the program (two data sets). | Both (parent / carer and program participant) | 10 | IV | Serious | ASD, Spina Bifida, CP, Speech or language delay and Developmental Delay | 1 year 4 months - 4 year 6 months (range)  Mean not reported. | Aquatic and land-based program | 1 session | 60 minutes | 4-week aquatic followed by a 4-week land program |
| da Silva et al., 2025 | Randomised controlled trial | Brazil | Performance and satisfaction with participant goals, participation pattern, physical literacy, family empowerment and physical activity level were assessed before the program, after the program and after follow up. | Program participant | 18 | II | Moderate | CP GMFCS (I-IV) | 6-12 years (range)  Sports Stars Brazil + Pathways and Resources for Engagement and Participation (PREP) group: 9.11 ± 2.02 years (mean ± SD)  Sports Stars Brazil only group: 8.33 ± 1.41 years | Sports Stars Brazil (soccer, handball, basketball and athletics)  PREP: Make goals, map out a plan, make it happen, measure process and outcomes and move forward | 1 session | 60 minutes | Sports Stars Brazil + PREP: 16 weeks (8 weeks Sports Stars Brazil then 8 weeks PREP)  Sports Stars Brazil only: 8 weeks |
| Sousa Junior et al., 2024 | Qualitative descriptive study | Brazil | Focus groups (4-6 participants in each) with questions guided by the F-words conducted after the program. | Parent / carer | 15 | IV | Moderate | CP (GMFCS I-II) | 6-12 years (range)  Mean not reported | Sports Stars Brazil (soccer, handball, basketball and athletics) | 1 session | 60 minutes | 8 weeks (different sport every 2 weeks) |
| Sousa Junior et al., 2025 | Randomised controlled trial | Brazil | Motor skills, body functions, physical activity levels, physical literacy and overall life participation were assessed before the program, after the program and after follow up. | Program participant | 38 | II | Low | CP (GMFCS I-II) | 6-12 years (range)  Sports Stars Brazil group: 8.11 ± 1.11 years (mean ± SD)  Control group: 8.4 ± 1.11 years (mean ± SD) | Sports Stars Brazil (soccer, handball, basketball and athletics) | 1 session | 60 minutes | 8 weeks (different sport every 2 weeks) |
| Sterba et al., 2002 | Pre-post test study design | USA | Functional independence and gross motor function measured 6-weeks before the start of the program, at the commencement of the program, during the program at Week 6 and 12, after the program, and 6-weeks after the completion of the program. | Program participant | 17 | IV | Serious | CP (GMFCS I-V) | 5-16 years (range)  9 years and 10 months ± 10 months (mean ± SD) | Horseback riding | 1 session | 60 minutes | 18 weeks |
| Tischler & O'Leary, 2022 | Case series | USA | Quality of life and participation in activities measures before and after the program. | Program participant | 4 | IV | Serious | CP, Down syndrome, muscular dystrophy, juvenile idiopathic arthritis, intellectual disability, autism spectrum disorder, or spinal muscular atrophy | 6-10 years (range)  7.25 ± 1.89 years (mean ± SD) | Yoga | 2 sessions | 60 minutes | 10 weeks |

Abbreviations – CP: Cerebral palsy, GMFCS: Gross Motor Function Classification System, USA: United States of America, ADHD: Attention Deficit Hyperactivity Disorder, SD: Standard deviation
